# Structural glycobiology – from enzymes to organelles

**DOI:** 10.1042/BST20241119

**Published:** 2025-01-31

**Authors:** Courtney J. Mycroft-West, Miron A. Leanca, Liang Wu

**Affiliations:** 1 The Rosalind Franklin Institute, Harwell Science & Innovation Campus, OX11 0QX, Didcot, UK; 2 Division of Structural Biology, Nuffield Department of Medicine, University of Oxford, OX3 7BN, Oxford, UK

## Abstract

Biological carbohydrate polymers represent some of the most complex molecules in life, enabling their participation in a huge range of physiological functions. The complexity of biological carbohydrates arises from an extensive enzymatic repertoire involved in their construction, deconstruction and modification. Over the past decades, structural studies of carbohydrate processing enzymes have driven major insights into their mechanisms, supporting associated applications across medicine and biotechnology. Despite these successes, our understanding of how multienzyme networks function to create complex polysaccharides is still limited. Emerging techniques such as super-resolution microscopy and cryo-electron tomography are now enabling the investigation of native biological systems at near molecular resolutions. Here, we review insights from classical *in vitro* studies of carbohydrate processing, alongside recent *in situ* studies of glycosylation-related processes. While considerable technical challenges remain, the integration of molecular mechanisms with true biological context promises to transform our understanding of carbohydrate regulation, shining light upon the processes driving functional complexity in these essential biomolecules.

## Introduction

**Figure 1:**
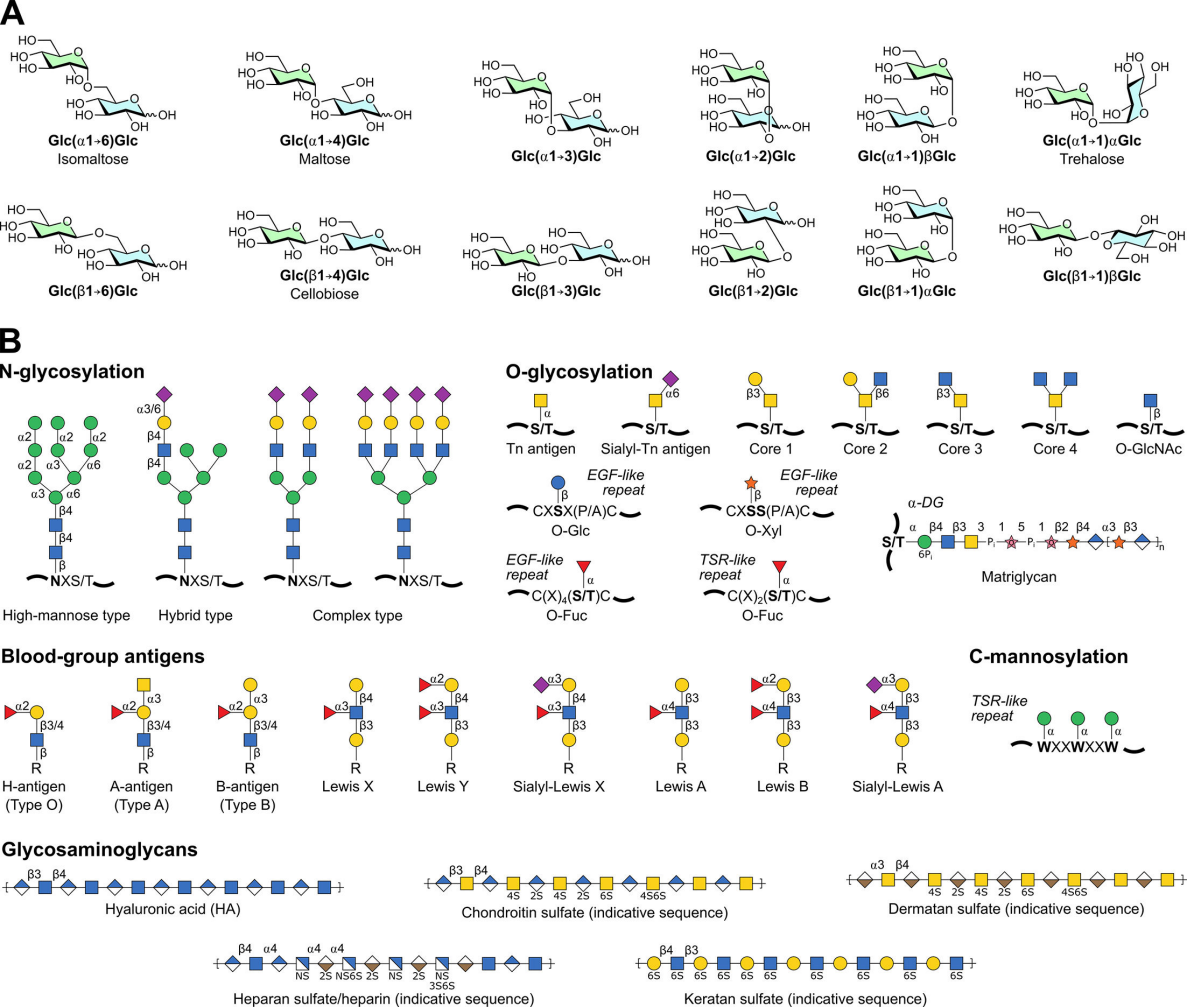
Molecular diversity of biological carbohydrates. (**A**) Regio- and stereo-isomeric combinations for a theoretical dimer of D-glucose. Eleven unique permutations are possible [Glc(α1→1)βGlc and Glc(β1→1)αGlc are equivalent], far exceeding the complexity of nucleic acids and proteins. (**B**) Selected glycan types found in eukaryotes, depicted in symbol nomenclature for glycans (SNFG) format [14]. Note that glycosaminoglycans are highly heterogenous and variable polymers – indicative sequences shown may not fully reflect natural complexity.

Carbohydrate (glycan/saccharide/sugar) polymers are among the most ubiquitous and important macromolecules in nature, used without exception across all kingdoms of life. Complex biological carbohydrates, either in free form or as conjugates of proteins, lipids, and metabolites, mediate countless structural [1–3], signalling [4–9], and metabolic functions [10–12], well beyond their colloquial designations as dietary energy sources. The wide-ranging roles of biological carbohydrates arise from their astonishing molecular diversity, enabling interactions with a huge range of partners [13]. Carbohydrate polymers are constructed from monosaccharide building blocks, which can link together in multiple regio- and stereoisomeric variations, producing combinatorial complexity far exceeding that of other biomolecules (Figure 1). Such complexity reflects an equally extensive enzymatic network tasked with regulating carbohydrate structures.

Precise enzymatic control of carbohydrates is of paramount importance in biology. Glycosidic linkages between monosaccharides must be made or broken with exquisite selectivity, within the context of larger oligo/polymeric assemblies. In turn, glycans containing multiple linkage types must be constructed by networks of enzymes acting in concert. Unlike nucleic acids or proteins, carbohydrates are not directly templated by genetics, meaning that multiple related structures can be produced by each pathway, depending on the functional interactions arising between enzymes and their substrates. Despite this lack of templating, cells nevertheless exert fine control over their glycosylation repertoire, enabling distinct motifs to be presented in a tissue and context specific manner [15–22].

Here, we review advances in structural glycobiology that have shaped our understanding of carbohydrate processing, particularly in eukaryotes. We cover mechanistic insights gained from *in vitro* studies of glycosylation and examine how these link to new *in situ* approaches that enable the examination of glycosylation within native biological systems.

### Carbohydrate processing enzymes – mechanistic considerations

Underscoring their importance, carbohydrate processing enzymes form a substantial part of the metabolic repertoire of all organisms, with 1–3% of the gene-coding content of any species typically dedicated to these functions [23,24]. The CAZy consortium has undertaken to classify all known Carbohydrate Active enZymes (CAZymes) within sequence-based families, providing a powerful framework on which to place mechanistic insights [25–27]. Within the CAZy classification, the glycoside hydrolases (GHs) and glycosyltransferases (GTs) make up the largest enzyme groupings, with 189 GH and 137 GT families annotated as of December 2024. These enzymes are responsible for the degradation and construction of biological carbohydrates, and accordingly, have spurred decades-long interest in understanding their functions. Structural techniques have contributed centrally to the mechanistic study of GHs and GTs, from Phillips’ groundbreaking elucidation of lysozyme by X-ray crystallography [28,29], to recent cryo-electron microscopy (cryo-EM) investigations of multicomponent GT complexes [30,31]. Mechanistic insights have, in turn, spurred the development of strategies for manipulation [32–35], supporting diverse applications across fundamental science, biomedicine and biotechnology [36–45].

### GH mechanisms

**Figure 2:**
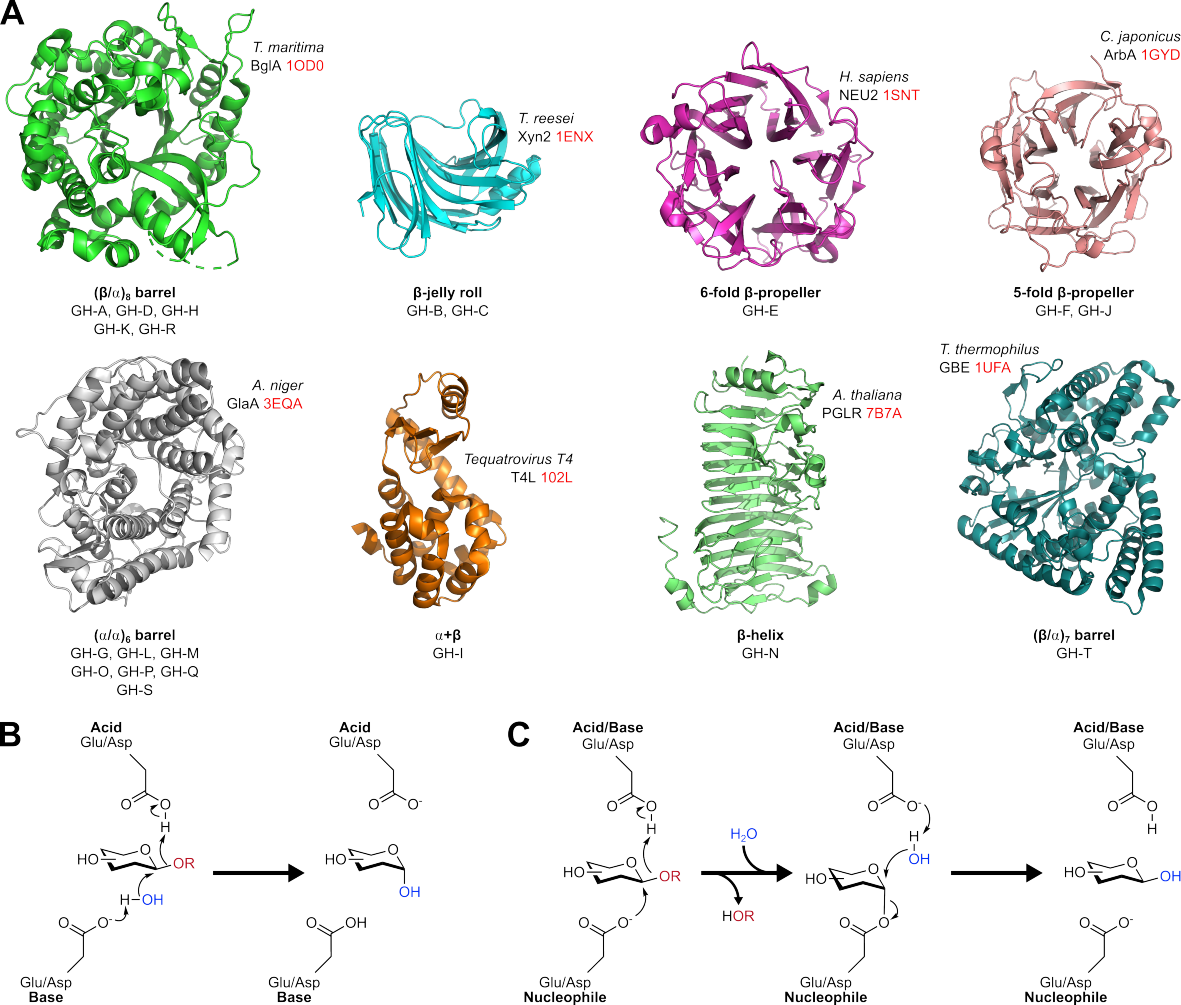
Structures and mechanisms of glycoside hydrolase enzymes. (**A**) Protein folds adopted by known GHs, with corresponding clans listed. One representative structure for each fold is shown [49–55]. PDB codes highlighted in red. (**B**) Schematic of the single S_N_2 displacement mechanism used by inverting GHs. (**C**) Schematic of the double-displacement mechanism used by retaining GHs, which results in net retention of anomeric stereochemistry after two S_N_2 attacks.

GHs are highly optimised molecular machines that hydrolyse glycosidic bonds [46,47], and represent the primary route for glycan catabolism across life. Mechanistically, GHs can be classified as inverting or retaining, based on the relative configurations of the glycosidic anomeric centre from substrate to product. Retaining GHs mediate hydrolytic cleavage with net retention of anomeric stereochemistry (i.e., α substrate to α product, or β to β) while inverting GHs flip anomeric stereochemistry (α to β, β to α). These GH activities are accommodated within a diverse range of protein folds, broadly conserved across ‘clans’ (i.e., superfamilies) within the CAZy scheme [48], (Figure 2a).

Most GHs utilise nucleophilic displacement to process their carbohydrate substrates, aided by conserved carboxylate (Asp/Glu) residues within the enzyme active site. Inverting GHs mediate hydrolysis using a single S_N_2-type attack, initiated by catalytic base-mediated deprotonation of an active site water, with loss of aglycone aided by protonation from a catalytic acid (Figure 2b). Conversely, retaining GHs typically employ double displacement mechanisms, in which initial S_N_2 attack on the substrate by a nucleophilic residue produces a transient glycosyl-enzyme intermediate, which is released by water after a second S_N_2 attack (Figure 2c). A smaller number of non-canonical mechanisms are also known, involving alternative nucleophiles [56], neighbouring group participation [57,58] or NAD^+^ cofactors [59–62].

### Glycosyltransferase mechanisms

**Figure 3:**
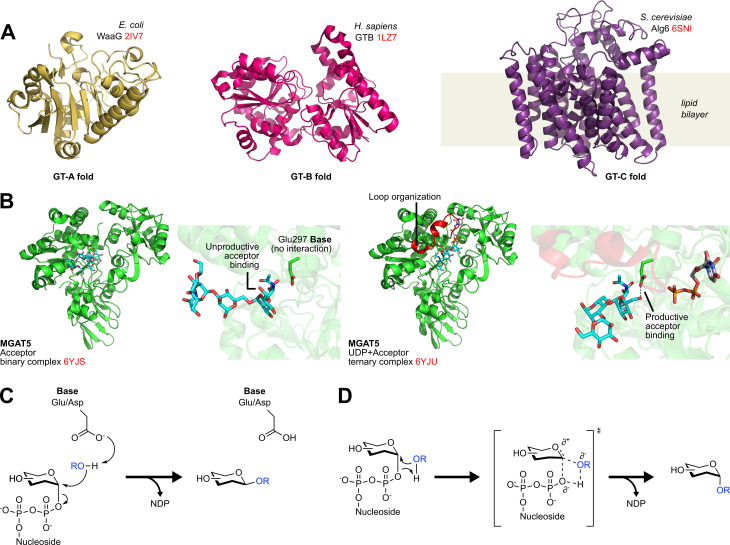
Structures and mechanisms of glycosyltransferase enzymes. (**A**) Protein folds adopted by known GTs. One representative structure for each fold is shown [65–67]. PDB codes highlighted in red. (**B**) GTs and allosteric modulation: MGAT5 in complex with acceptor oligosaccharide alone or acceptor + UDP. In the absence of UDP, the acceptor oligosaccharide adopts an inert pose with no interaction to the catalytic base. A loop organised upon UDP binding (red) alters the acceptor binding pose to induce catalytically productive interactions [68]. (**C**) Schematic of canonical S_N_2 displacement mechanism in inverting GTs. Note the similarity to inverting GHs. (**D**) Schematic of S_N_i-like mechanism for retaining GTs involving front-face transfer.

GTs catalyse the formation of new glycosidic linkages, transferring carbohydrate units from activated glycosyl donors onto specific acceptor molecules. In contrast to GHs, GT enzymes display much less structural diversity, with only three clans currently classified. The GT-A and GT-B clans largely comprise soluble proteins adopting α/β/α sandwich or paired-Rossman folds, respectively, and use sugar-nucleotide (‘Leloir’) substrates as glycosyl donors [63]. In contrast, clan GT-C exclusively comprises integral multipass transmembrane (TM) proteins, which use sugar-phosphate linked lipid (‘non-Leloir’) substrates as donors [64], (Figure 3a). Note that while GT-A and GT-B domains are themselves typically soluble, they can still link to membrane domains as part of broader protein structures, as observed for cellulose [69–71], and hyaluronan synthases (both GT-A) [72], wherein enzyme products are conjugated to closely abutting TM channels. Many GTs also display allosteric shifts upon the binding of either glycosyl donor [68,73–76] or acceptor substrate [77], such that reactive binding modes only occur upon the formation of a complete ternary complex. Care must, therefore, be exercised in the generation and interpretation of GT structures for mechanistic study (Figure 3b).

Like GHs, GTs can be classified as retaining or inverting, depending on the stereochemical relationship between the substrate and product. Inverting GTs operate analogously to inverting GHs, using a single S_N_2-type displacement to form glycosidic bonds, driven by base-mediated deprotonation of the acceptor substrate (Figure 3c). In contrast, most retaining GTs are proposed to operate *via* non-nucleophilic S_N_i-type front-face transfer, involving the constrained approach of the acceptor from the same ‘face’ as the glycosyl donor bond [78–81], (Figure 3d). While some unusual double displacement GT reactions have recently been identified for retaining Kdo-transferases involved in bacterial cell wall construction [82,83], the extent to which these mechanisms operate across other GT families remains to be determined.

Special consideration must be given to GT-C enzymes, which historically had fewer structures reported prior to the wider adoption of cryo-EM for studying membrane proteins. Classical GT-C folds comprise a core region containing seven conserved TM helices, followed by a variable number of additional helices, with the essential catalytic base residing in an extended loop following TM helix 1 (all known GT-C enzymes are inverting) [30,64,65,77,84–86]. Interestingly, an alternative arrangement of ten TM helices has recently been noted for bacterial GT-Cs RodA [87] and WaaL [88], highlighting potentially undiscovered structural diversity within this poorly characterised clan.

For all GT-Cs, binding of lipid-linked glycosyl donor substrates occurs within hydrophobic TM cavities, leading to placement of sugar headgroups near the glycosyl acceptor and catalytic base, in line with canonical inverting GT transfer, with occasional variations, e.g., a His base in WaaL rather than Asp/Glu [88]. Perhaps more intriguing is the ability of some GT-C enzymes to glycosylate poorly nucleophilic acceptors. The eukaryotic oligosaccharyltransferases OST-A/B [30,65], and bacterial homologues such as PglB, transfer glycans onto the unreactive amide nitrogen of Asn sidechains, which is putatively achieved *via* H-bond-mediated twisting of the amido nitrogen, breaking the conjugated π-system that otherwise dampens nucleophilicity [89]. Separately, CMTs, which catalyse tryptophan C-mannosylation, are proposed to function *via* an S_N_Ar-type mechanism, with initial electrophilic substitution at tryptophan C2 closely followed by base-mediated deprotonation to restore aromaticity [77].

## Understanding glycosylation networks *in vitro*

**Figure 4:**
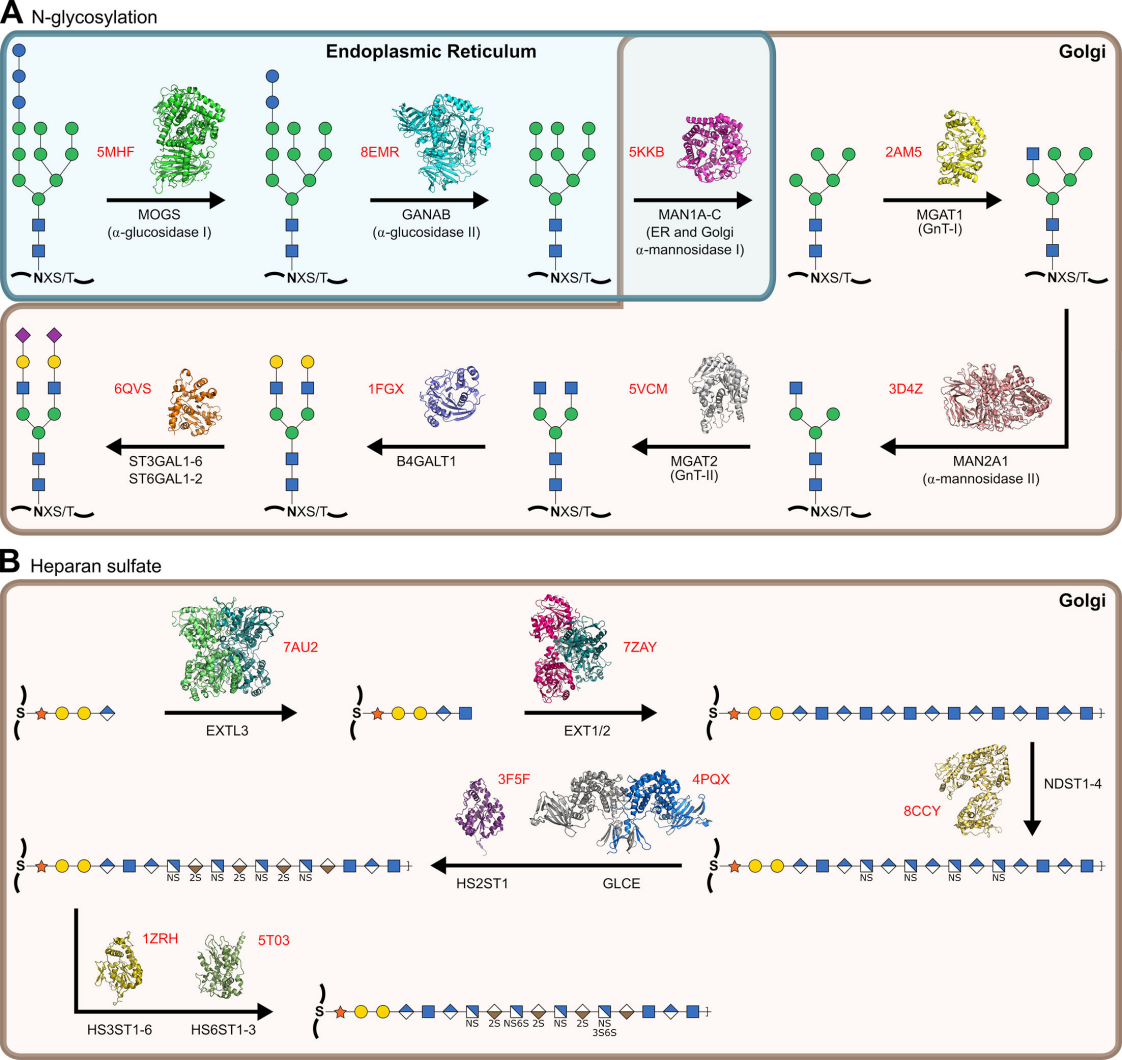
Near-complete structural characterisation of selected glycosylation pathways. (**A**) Protein N-glycan processing within the ER and Golgi to form complex-type biantennary glycans, with structures of responsible enzymes shown [98–105]. For clarity, processing pathways to form hybrid, bifurcated or tri-/tetra-antennary complex glycans have been omitted. (**B**) Heparan sulfate biosynthesis within the Golgi, from the first committed step after formation of the core tetrasaccharide, with structures of responsible enzymes shown [106–111]. PDB codes highlighted in red.

Although many simple glycan motifs, such as protein modification by O-GlcNAc [90], C-mannose [91] or O-fucose [92], play important physiological functions, e.g., regulation of transcription and epigenetics by nuclear O-GlcNAc [93–97], most biological carbohydrates are considerably more complex (Figure 1). These complex glycans are built by multi-step biosynthesis pathways, wherein the product of one enzyme forms the substrate of one or more downstream enzymes. Structural studies have now made considerable headway into glycosylation processes, with major pathways such as N-glycosylation and heparan sulfates (HS) nearing full characterisation, enabling understanding of how their activities evolve as glycan products mature (Figure 4). As an example, recent structures of EXTL3 and EXT1/2 inform upon the initial construction of the HS backbone by non-processive polymerisation [106,112], with further HS modification by deacetylation, epimerisation and sulfation rationalised by the structures of NDST1 [107], GLCE [108] and O-sulfotransferases [110,113–115], respectively.

Despite our considerable understanding of individual glycosylation enzymes, many aspects of overall carbohydrate regulation still remain unclear. In particular, *in vitro* characterisation cannot explain the diverse but regulated heterogeneity of complex biological carbohydrates. Without direct templating, glycosylation is likely controlled by the coordinated and controlled transfer of substrates between successive enzymes, directing certain motifs to be constructed whilst minimizing off-target reactions. Understanding this functional interplay between glycosylation enzymes, and the mechanisms that link catalysis with substrate transfer, can only be achieved by studying native systems.

## Towards understanding (eukaryotic) glycosylation *in situ*

For eukaryotes, a major proportion of carbohydrate processing occurs within the secretory ER-Golgi network, involving the action of resident GHs and GTs upon nascent glycoproteins as they transit towards their destinations (Figure 4). Partitioning of glycosylation enzymes along the Golgi stack has long been understood, with most early-acting enzymes residing in the *cis-*Golgi and later enzymes in the *medial-* and *trans-*Golgi, providing some hierarchical control over their successive activities [116–118].

Considerable evidence also implicates ‘kin-recognition’ as another mechanism for regulating glycosylation, whereby enzymes within a pathway form homo- or heteromeric complexes within the ER-Golgi to coordinate their functions. Early work by Nilsson et al. used relocalisation tags to probe physical relationships between human glycosylation enzymes. By grafting the ER-directing sequence of p33 onto *medial*-Golgi enzymes MGAT1 or MAN2A1**,** which act successively during N-glycosylation, the non-tagged partner could also be relocated. Conversely, p33 tagging of the *trans*-Golgi resident β-galactosyltransferase failed to relocate either MGAT1 or MAN2A1, suggesting specific coordination between the former pair [119]. Many similar relationships have now been identified across yeast [120], plants [121] and human [122] cells. Notably, several examples of kin-recognition have been reported within the biosynthesis pathway of the glycosaminoglycan HS, with EXT1 and EXT2 known to form a stable heterodimer [106,112,123], EXTL3 forming a homodimer [111], and EXT2 and NDST1 [124], and GLCE and HS2ST1 [125,126] also postulated to interact (Figure 4b). Based on the rapidity of HS construction in mouse mastocytoma fractions (minutes) [127], it has been theorised that HS biosynthesis enzymes may form a functional supercomplex, dubbed the GAGosome, responsible for coordinated construction of this polysaccharide [128]. However, no clear evidence for such an assembly has yet emerged. Indeed, with the exception of some obligate dimers [106,111,112], almost nothing is known about how kin-recognition in general may operate within the ER-Golgi, reflecting the likely transient nature of these interactions [122,129] and their lability outside of native environments.

Recently, super-resolution microscopy studies have started to tackle the complexities of Golgi function, enabling prior observations of interactions to be contextualised within living cells [130–134]. From imaging, the main organisational component of the Golgi complex appears to be the so-called ‘Golgi unit’, which broadly corresponds to the cisternae structures commonly associated with this organelle. Individual Golgi units are bounded by markers including GPP130, Golgin84 and Giantin, and connect to adjacent units *via* tubules, forming a broader Golgi ribbon superstructure. Within each Golgi unit, glycosylation enzymes are distributed to punctate zones, with those catalysing earlier-stage reactions occupying more ‘peripheral’ locations compared with later-stage enzymes, indicating that lateral as well as transverse enzyme partitioning may operate to regulate carbohydrate biosynthesis. Golgi units are also observed to be highly dynamic, with both splitting and fusion occurring over timeframes of minutes, potentially facilitating the even distribution of enzymes [134]. It is thus clear that subcellular imaging can deliver powerful insights into Golgi glycosylation processes. However, the spatial resolutions offered by super-resolution light microscopy still fall below that required for directly visualizing enzyme interactions. More precise molecular identification requires resolutions currently only afforded by electron imaging.

## Cryo-ET – direct visualisation of native biology

The recent emergence of cryo-electron tomography (cryo-ET) and associated techniques for biological imaging has provided a powerful toolkit for the study of intracellular organisation. Like the application of cryo-EM for single particle analysis, cryo-ET uses transmission electron microscopy (TEM) to image vitrified samples at near-atomic resolutions. Whereas single-particle data typically comprise many thousands of images of a given molecule for averaging, cryo-ET focuses on imaging a single site at multiple tilts, enabling subsequent three-dimensional reconstruction of a volume of interest.

Although cryo-ET projects remain significant undertakings, several recent advances have greatly increased the accessibility of this technique for interrogating biological environments. One of the most fundamental limitations of cryo-ET arises from the low mean free path (i.e., penetrating ability) of electrons, which limits the thickness of samples that can be studied to ~200 nm or less [135]. Thus, while viruses [136–139], vesicles [140], small bacteria [141,142] and cell peripheries [143–145] are readily imaged by cryo-ET, thicker specimens such as cell bodies or tissues must first be thinned to electron transparency. Diamond knife cryo-sectioning has historically been used to process samples to required thicknesses but can cause sample compression and crevassing artefacts that distort resulting images [146]. The advent of gallium [147], and more recently plasma-based [148,149] focussed ion beam (FIB) milling, coupled to scanning electron microscopy (FIB-SEM), has now largely replaced cryo-sectioning for biological samples, enabling cryo-ET-compatible lamellae to be rapidly prepared from cells with high throughput and minimal distortion [150]. By combining FIB milling with cryo-lift out strategies, sections of tissue or even whole organisms are now within reach [151–155]. Further integration of fluorescence microscopy also enables correlated light and electron microscopy (CLEM) approaches, whereby regions of interest can be targeted for FIB milling and cryo-ET based on incorporated fluorescent markers (e.g. an ER or Golgi stain to study glycosylation), thus bridging the imaging scales traditionally occupied by cellular and molecular biology [156–158].

A second major challenge for cryo-ET is the poor contrast arising from low-dose imaging of sensitive biological samples [159], compounded by the geometric increase in lamella thickness that occurs at higher tilts [160]. As with single-particle cryo-EM, recent developments in field emission guns, direct electron detectors, energy filters [161] and phase plates [162,163] have now substantially improved the data quality that can be obtained from vitrified biological samples. New processing methods have also streamlined workflows [164–166], with recent machine-learning tools significantly enhancing the speed and accuracy of cryo-ET annotations [167–171]. In favourable cases, with abundant well-resolved particles, near-atomic structures are now achievable using subtomogram averaging, wherein multiple copies of a particle are extracted from tomograms, aligned, and averaged to higher resolution. Stunning recent reconstructions of ribosomes at ~3–4 Å directly demonstrate the potential of *in situ* elucidation [141–143]. Where high resolutions are not possible, lower resolution volumes can still be used to dock coordinates from cryo-EM or X-ray crystallography, linking molecular models to *in situ* context. We direct interested readers to excellent recent reviews of this rapidly emerging field, including technical considerations not possible to cover here [172–174].

## Cryo-ET studies of the ER-Golgi pathway

**Figure 5:**
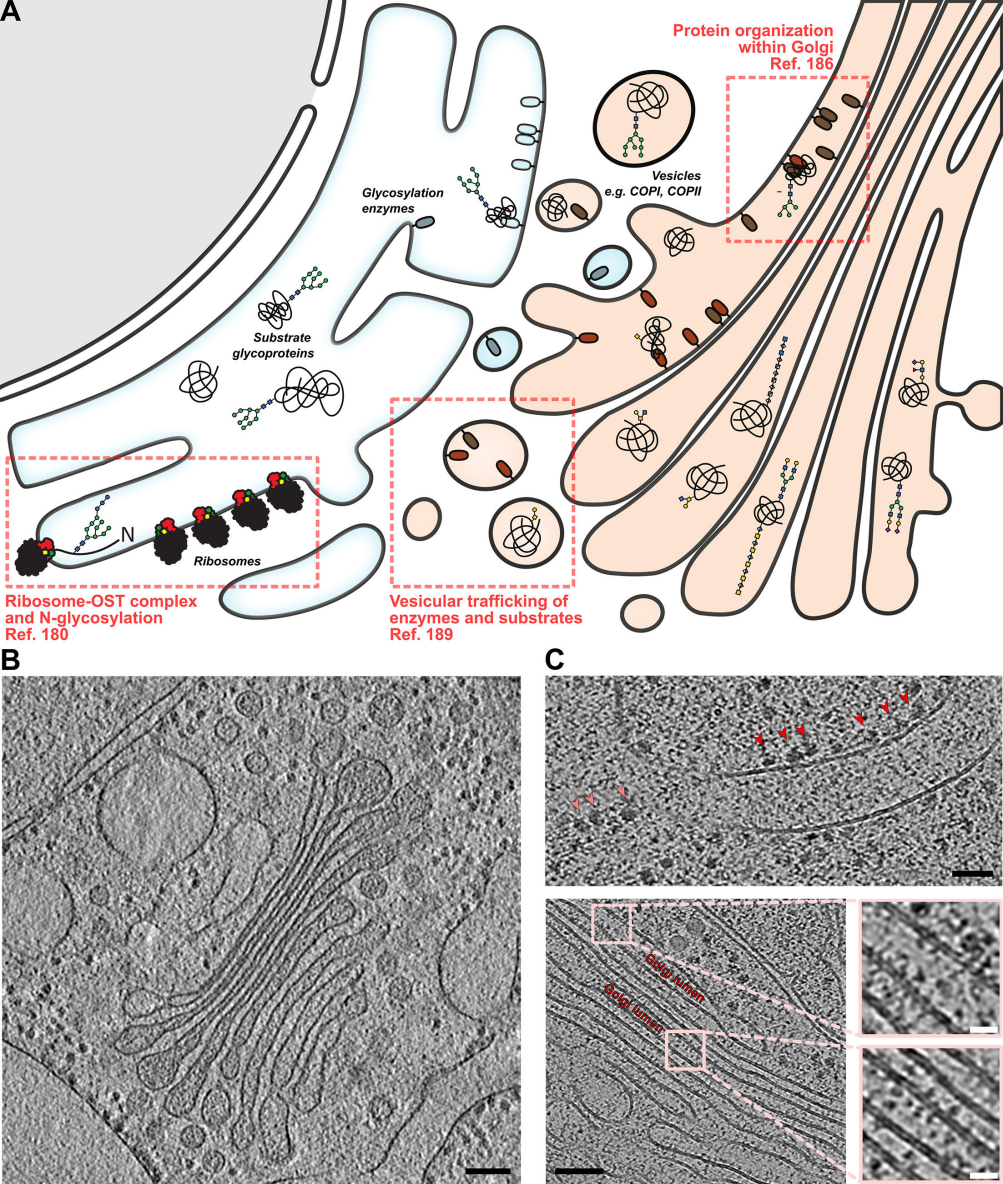
*In situ* glycosylation pathways and their examination by cryo-ET. (**A**) Schematic of eukaryotic glycosylation within the ER-Golgi pathway. Nascent protein substrates are modified by GHs and GTs as they transit through the ER and successive Golgi cisternae. ‘Kin-recognition’ between enzymes may facilitate control, driving the production of certain glycan motifs, despite the overall non-templated nature of glycosylation. Boxes reference recent cryo-ET studies of systems related to glycan processing. (**B**) Tomogram of Golgi apparatus from *C. reinhardii*. Stack morphology of the organelle is clearly apparent, alongside COP vesicles that distribute cargo between cisternae. Adapted from EMD-3977 [175] (**C**) Top – tomogram of HeLa ER membrane and surroundings, showing attachment of ribosomes (red arrows), alongside detached cytosolic ribosomes (pink). Bottom – tomogram of HeLa Golgi at higher magnification. Defined densities are visible on the luminal membrane face, which may (in part) correspond to enzymes involved in glycosylation. From authors’ own work. Black bars – 100 nm. White bars – 20 nm.

Due to substantial challenges (see below), studies of carbohydrate processing by cryo-ET are still in their infancy. However, several recent reports have highlighted the use of this technique to investigate eukaryotic ER-Golgi networks, shedding light on some key processes that impact glycosylation (Figure 5).

### Native studies of the ER

A central function of the eukaryotic ER is the initiation of N-glycosylation, whereby a Glc_3_Man_9_GlcNAc_2_ oligosaccharide is transferred from dolichol-linked donors onto **N**XS/T sequons in nascent protein chains. This process is carried out by the oligosaccharyltransferase complexes OST-A/B, which associate with the ribosome, SEC61 translocon and translocon-associated protein (TRAP) complex to co-translationally (OST-A) or post-translationally (OST-B) modify peptides as they enter the ER lumen [65,176]. Seminal studies by Förster et al. have used cryo-ET to study the ribosome-translocon supercomplex in semi-purified ER microsomes, which provide a simpler system for analysis compared with whole cells while still maintaining membrane context. Although early efforts from 2011 achieved structures at only modest (~31 Å) resolutions, spatial relationships between ribosomes and the TRAP and OST complexes could still be identified, enabling both the stoichiometry and organisation of this macromolecular assembly to be verified [177–179]. With modern technical advances, the same team has recently achieved cryo-ET reconstructions of ER ribosomes to 4–10 Å, enabling at least ten decoding states across the ribosomal translation cycle to be classified, and polysomal networks to be traced [180]. Importantly for glycosylation, four ribosome-bound translocon states could also be classified, with 69% representing the SEC61-TRAP-OST-A supercomplex, enabling elucidation of a ~ 4.2 Å structure. The resulting model of the native translocon has enabled hypotheses regarding the essential role of TRAP in glycosylating proteins with weaker signal peptides [181]. Putatively, nascent polypeptides entering the ER are likely to encounter and push upon the TRAP α-subunit, which can interact with SEC61α to allosterically open its hydrophobic lateral gate, easing the entry of peptides into the SEC61 channel and towards glycosylation by OST-A.

### Native studies of the Golgi

Electron micrographs of fixed Golgi have been reported since at least the 1950s [182–184], and vitrified cryo-sectioned Golgi since the 2000s [185], unveiling broad aspects of organellar morphology. Detailed cryo-ET imaging of Golgi from FIB-milled lamellae was first reported by Engel et al. in 2015, using the marine algae *Chlamydomonas reinhardii*, revealing a classical stack-like organisation, with progressively narrowing cisternae from *cis* to *trans* (Figure 5b) [186]. Intriguingly, close examination of the *Chlamydomonas trans*-Golgi revealed regularly arrayed densities, which were hypothesised to correspond to GTs, based on similarities in size to Golgi-resident FUT6, GMII and α3GalT. Whilst spatial organisation of enzymes within the Golgi arrays is a compelling hypothesis, the extent to which these densities truly correspond to GTs remains unverified, as does the generality of these observations beyond *Chlamydomonas*. Notably, intracisternal arrays have not been observed in other Golgi tomograms, such as those from mammalian HeLa or INS-1E cells, which also exhibit different organellar morphologies (Figure 5c) [187]. It is possible that the arrays observed in *Chlamydomonas* Golgi represent structural proteins, rather than metabolic enzymes. Characterisation of the Golgi across more species and cell types will be key to establishing general vs. specific features of this organelle, including the nature of macromolecular organisation within its cisternae.

### Native studies of vesicular transport

Another major factor affecting eukaryotic glycosylation is the distribution of enzymes within Golgi [133], which depends on the dynamic network of COPI (retrograde) and COPII (anterograde) vesicles that move cargo throughout the organelle [188]. *In situ* reconstructions of *Chlamydomonas* COPI vesicles were reported in 2017 by Briggs et al. [175], highlighting the trimeric structures of their coat proteins, which closely matched vesicles previously generated *in vitro* using mouse proteins [189]. Interestingly, *in situ* COPI vesicles contained several additional densities on their luminal faces not seen *in vitro*, likely corresponding to bound cargo or cargo receptors, enabling some insights into trafficking. Further integration with functional studies will be needed to resolve the mechanisms of vesicular transport and understand how these influence enzyme distribution throughout the Golgi.

## Challenges for studying glycosylation by cryo-ET

### Size limitations

A major challenge studying glycosylation by cryo-ET is the small size of most relevant enzymes (typically 50–200 kDa; ~10 nm), which lies towards the lower bound of what can be easily identified within a crowded intracellular milieu. Consequently, relatively few insights have been made into glycosylation-specific processes, despite detailed tomograms of the ER-Golgi being available for nearly a decade. It is clear that improved tools and methodologies are needed to annotate complex cryo-ET datasets.

The principal strategy for locating smaller proteins by cryo-ET is to colocalise a recognisable marker at or near the site of interest. Historical efforts have widely employed Au nanoparticles to label tomograms, due to the inertness of Au and its high atomic number compared with biological elements, which creates strong signals under TEM imaging regimes. Au nanoparticles conjugated to proteins or antibodies have been used to study diverse phenomena by cryo-ET, including transport across the nuclear pore [190], viral biogenesis [191,192], thylakoid photosystems [193] and growth factor trafficking [194]. Although large Au nanoparticles can obscure biological features of interest in tomograms, Young et al. recently demonstrated that 1.4 nm Au clusters are sufficiently non-intrusive to support sub-tomogram averaging of ribosomes and nucleosomes [195]. Combined with ever-improving technologies to raise custom binders, e.g., nanobodies [196], the ability to tag any cellular component is now theoretically within reach, although delivery of abiotic nanoparticles into cells still remains non-trivial. Some reported strategies here include permeabilisation of cell membranes using streptolysin O [195] or the uptake of BSA-conjugated Au *via* the endo-lysosomal pathway [197]. Both approaches are likely limited by toxicity and can access only certain cellular compartments, limiting their general applicability.

Ultimately, the development of universal genetically encodable tags is likely to prove transformational for cryo-ET, similar to how fluorescent proteins revolutionised light microscopy. Encodable metallothionein [198] or ferritin-based [199,200] tags, fused to proteins of interest, have been demonstrated to improve cryo-ET contrast *via* their ability to sequester multiple transition metal ions with high affinity. However, biological systems studied using these tags must be enriched with relevant metals to enable loading, causing potential issues with toxicity or incompatibility. DNA origami labels have also shown promise as distinctive markers, which can readily be targeted to proteins of interest *via* hybridisation to a suitable RNA aptamer [201]. Although these DNA labels are not expressible in a strict biological sense and are limited to use on cell surfaces, the use of distinctive nucleic acid shapes highlights a potentially powerful strategy that may be further developed. Another elegant approach was recently reported by Fung et al., employing genetically encoded encapsulin multimers that conditionally bind GFP in the presence of a rapamycin analogue [202]. Use of these ‘GEM-tags’ drives colocalisation of a 25–45 nm icosahedral particle next to GFP-labelled proteins of interest, supporting CLEM strategies, as well as facile target detection in tomograms by template-picking [203]. The main drawback of the GEM system appears to be the multimeric nature of encapsulin itself, which may induce multimerisation of targets, perturbing them from their native locations.

### Temporal limitations

The dynamic nature of glycosylation represents another significant hurdle for cryo-ET, which can only provide static snapshots of vitrified biological samples. Thus, while many relevant enzyme–substrate and enzyme–enzyme interactions may be identified from ER-Golgi tomograms, understanding how these link together over the course of glycan biosynthesis requires methods such as super-resolution microscopy, which has already delivered important insights into Golgi dynamics [31,32,35]. CLEM approaches that combine cryo-ET with live-cell imaging can provide powerful additional context that enriches both techniques, enabling molecular-level characterisations of key events to be placed within their correct temporal order [204]. One potential strategy to achieve this goal is the coupling of (super-resolution) light microscopy platforms with rapid cryo-fixation, enabling vitrification at exact time points of interest identified by fluorescence, supporting subsequent high-resolution dissection by cryo-ET [205].

## Outlook

Although most studies of glycosylation to date have largely focussed on mechanisms of individual enzymes, the frontier increasingly lies in resolving how these enzymes operate together within their native environments. The ability of cryo-ET to visualise *in situ* biology at high resolution renders it a powerful tool to study multicomponent systems such as enzyme networks. Despite this promise, the study of carbohydrate regulation by cryo-ET remains in its infancy, largely owing to the difficulties of identifying small enzymes and dynamic processes within the ER-Golgi network. Most cryo-ET studies of ER-Golgi to date have either examined aspects of organellar or vesicular morphology [175,186–188], or larger entities such as ribosomes on ER membranes [180]. While underlying technical challenges are likely to persist for some time, the advent of improved imaging and processing methods, coupled with labelling and CLEM strategies, represent exciting developments for the future. Resolving dynamic patterns of enzyme organisation within ER-Golgi by live-cell and cryo-ET imaging, even at modest resolutions, will shed considerable light on how these organelles operate to regulate complex glycans. In time, elucidating *in situ* structures will also reveal the molecular nature of kin interactions between enzymes, allowing us to see how their mechanisms fit within the context of broader functional assemblies.

The rich history of structural glycobiology has provided the field with many powerful insights into the function of carbohydrate-processing enzymes. We envision that the advent of *in situ* methods will enable classical insights to be placed within the complex nature of the cell, bringing our understanding of carbohydrate regulation from the molecular scale to organelles and beyond.

**Perspectives**

- Complex carbohydrates represent some of the most important biological macromolecules across life. Precise enzymatic regulation of carbohydrates is a critical process that underpins myriad biological functions, with an impact across health and disease.
- Over the past decades, the mechanisms of carbohydrate processing enzymes have stimulated intense discussion and investigation. The identification of conserved molecular mechanisms underpinning carbohydrate construction and deconstruction, by glycosyltransferases and glycoside hydrolases respectively, highlights the fruits of such efforts.
- Complex carbohydrates are constructed from networks of enzymes with no direct template. The frontier is increasingly understanding how these many enzymes coordinate within a complex intracellular milieu in a fashion that enables control over their products.

## Abbreviations

CAZymes: Carbohydrate Active enZymes
CLEM: Correlated Light and Electron Microscopy
FIB: focussed ion beam
FIB-SEM: FIB milling coupled with scanning electron microscopy
GHs: gycoside hydrolases
GTs: glycosyltransferases
HS: heparan sulfates
TEM: transmission electron microscopy
TM: transmembrane
TRAP: translocon-associated protein
cryo-ET: cryo-electron tomography

## References

[1] Vincent J.F (1999). From cellulose to cell. J. Exp. Biol..

[2] Moussian B (2019). Chitin: structure, chemistry and biology. Adv. Exp. Med. Biol..

[3] Naba A (2024). Mechanisms of assembly and remodelling of the extracellular matrix. Nat. Rev. Mol. Cell Biol..

[4] Pellegrini L., Burke D.F., DelftA Mulloy B., Blundell T.L (2000). Crystal structure of fibroblast growth factor receptor ectodomain bound to ligand and heparin. Nat. New Biol..

[5] Gray A.L., Karlsson R., Roberts A.R.E., Ridley A.J.L., Pun N., Khan B (2023). Chemokine CXCL4 interactions with extracellular matrix proteoglycans mediate widespread immune cell recruitment independent of chemokine receptors. Cell Rep..

[6] Poole J., Day C.J., Itzstein M.vonPaton J.C., Jennings M.P (2018). Glycointeractions in bacterial pathogenesis. Nat. Rev. Microbiol..

[7] Ströh L.J., Stehle T (2014). Glycan engagement by viruses: receptor switches and specificity. Annu. Rev. Virol..

[8] Clausen T.M., Sandoval D.R., Spliid C.B., Pihl J., Perrett H.R., Painter C.D (2020). SARS-CoV-2 Infection depends on cellular heparan sulfate and ace2. Cell.

[9] Long J.S., Mistry B., Haslam S.M., Barclay W.S (2019). Host and viral determinants of influenza A virus species specificity. Nat. Rev. Microbiol..

[10] Yang Y., Fu M., Li M.-D., Zhang K., Zhang B., Wang S (2020). O-GlcNAc transferase inhibits visceral fat lipolysis and promotes diet-induced obesity. Nat. Commun..

[11] Li M.-D., Vera N.B., Yang Y., Zhang B., Ni W., Ziso-Qejvanaj E (2018). Adipocyte OGT governs diet-induced hyperphagia and obesity. Nat. Commun..

[12] Hanover J.A., Krause M.W., Love D.C (2012). Bittersweet memories: linking metabolism to epigenetics through O-GlcNAcylation. Nat. Rev. Mol. Cell Biol..

[13] Werz D.B., Ranzinger R., Herget S., Adibekian A., Seeberger P.H (2007). Exploring the Structural Diversity of Mammalian Carbohydrates (“Glycospace”) by Statistical Databank Analysis. ACS Chem. Biol..

[14] Neelamegham S., Aoki-Kinoshita K., Bolton E., Frank M., Lisacek F., Lütteke T (2019). Updates to the Symbol Nomenclature for Glycans guidelines. Glycobiology.

[15] Wallace E.N., West C.A., McDowell C.T., Lu X., Bruner E., Mehta A.S (2024). An N-glycome tissue atlas of 15 human normal and cancer tissue types determined by MALDI-imaging mass spectrometry. Sci. Rep..

[16] Netelenbos T., Dräger A.M., Van het hof B., Kessler F.L., Delouis C., Huijgens P.C (2001). Differences in sulfation patterns of heparan sulfate derived from human bone marrow and umbilical vein endothelial cells. Exp. Hematol..

[17] Medzihradszky K.F., Kaasik K., Chalkley R.J (2015). Tissue-Specific Glycosylation at the glycopeptide level. Mol. Cell Proteomics.

[18] Lyon M., Deakin J.A., Gallagher J.T (1994). Liver heparan sulfate structure. A novel molecular design. J. Biol. Chem.

[19] Lindahl B., Eriksson L., Lindahl U (1995). Structure of heparan sulphate from human brain, with special regard to Alzheimer’s disease. Biochem. J..

[20] Gudelj I., Lauc G., Pezer M (2018). Immunoglobulin G glycosylation in aging and diseases. Cell. Immunol..

[21] Johnson C.E., Crawford B.E., Stavridis M., Ten Dam G., Wat A.L., Rushton G (2007). Essential alterations of heparan sulfate during the differentiation of embryonic stem cells to Sox1-enhanced green fluorescent protein-expressing neural progenitor cells. Stem Cells.

[22] Pinho S.S., Reis C.A (2015). Glycosylation in cancer: mechanisms and clinical implications. Nat. Rev. Cancer.

[23] Davies G.J., Gloster T.M., Henrissat B (2005). Recent structural insights into the expanding world of carbohydrate-active enzymes. Curr. Opin. Struct. Biol..

[24] El Kaoutari A., Armougom F., Gordon J.I., Raoult D., Henrissat B (2013). The abundance and variety of carbohydrate-active enzymes in the human gut microbiota. Nat. Rev. Microbiol..

[25] Drula E., Garron M.-L., Dogan S., Lombard V., Henrissat B., Terrapon N (2022). The carbohydrate-active enzyme database: functions and literature. Nucleic Acids Res..

[26] Cantarel B.L., Coutinho P.M., Rancurel C., Bernard T., Lombard V., Henrissat B (2009). The Carbohydrate-Active EnZymes database (CAZy): an expert resource for Glycogenomics. Nucleic Acids Res..

[27] Lombard V., Golaconda Ramulu H., Drula E., Coutinho P.M., Henrissat B (2014). The carbohydrate-active enzymes database (CAZy) in 2013. Nucleic Acids Res..

[28] Lake C., Mair C., North G.A., Phillips D.C., Sarma V.R (1967). On the conformation of the hen egg-white lysozyme molecule. Proc. R. Soc. Lond., B, Biol. Sci..

[29] Blake C.C., Johnson L.N., Mair G.A., North A.C., Phillips D.C., Sarma V.R (1967). Crystallographic studies of the activity of hen egg-white lysozyme. Proc. R. Soc. Lond., B, Biol. Sci..

[30] Wild R., Kowal J., Eyring J., Ngwa E.M., Aebi M., Locher K.P (2018). Structure of the yeast oligosaccharyltransferase complex gives insight into eukaryotic N-glycosylation. Science.

[31] Ramírez A.S., Kowal J., Locher K.P (2019). Cryo-electron microscopy structures of human oligosaccharyltransferase complexes OST-A and OST-B. Science.

[32] McCarter J.D., Yeung W., Chow J., Dolphin D., Withers S.G (1997). Design and Synthesis of 2‘-Deoxy-2‘-Fluorodisaccharides as Mechanism-Based Glycosidase Inhibitors That Exploit Aglycon Specificity. J. Am. Chem. Soc..

[33] Delbrouck J.A., Chêne L.P., Vincent S.P (2019). In In Fluorine in Life Sciences: Pharmaceuticals, Medicinal Diagnostics, and Agrochemicals.

[34] Linclau B., Ardá A., Reichardt N.-C., Sollogoub M., Unione L., Vincent S.P (2020). Fluorinated carbohydrates as chemical probes for molecular recognition studies. Current status and perspectives. Chem. Soc. Rev..

[35] McCarter J.D., Withers S.G (1996). 5-Fluoro Glycosides: a new class of mechanism-based Inhibitors of both α- and β-Glucosidases. J. Am. Chem. Soc..

[36] Schröder S.P., McGregor N.G.S., Rowland R.J., Moroz O., Blagova E (2019). Dynamic and functional profiling of xylan-degrading enzymes in aspergillus secretomes using activity-based probes. ACS Cent. Sci..

[37] Chauvigné-Hines L.M., Anderson L.N., Weaver H.M., Brown J.N., Koech P.K., Nicora C.D (2012). Suite of activity-based probes for cellulose-degrading enzymes. J. Am. Chem. Soc..

[38] Armstrong Z., Lit V.A.J., Barash U., Ruijgrok G., Boyango I (2022). Mechanism-based heparanase inhibitors reduce cancer metastasis in vivo. Proc. Natl. Acad. Sci. U.S.A..

[39] Kim J.-H., Resende R., Wennekes T., Chen H.-M., Bance N., Buchini S (2013). Mechanism-based covalent neuraminidase inhibitors with broad-spectrum influenza antiviral activity. Science.

[40] Witte M.D., Kallemeijn W.W., Aten J., Li K.-Y., Strijland A., Donker-Koopman W.E (2010). Ultrasensitive in situ visualization of active glucocerebrosidase molecules. Nat. Chem. Biol..

[41] Males A., Raich L., Williams S.J., Rovira C., Davies G.J (2017). Conformational Analysis of the Mannosidase Inhibitor Kifunensine: A Quantum Mechanical and Structural Approach. Chembiochem.

[42] Schröder S.P., Wu L., Artola M., Hansen T., Offen W.A., Ferraz M.J (2018). Gluco-1 H-imidazole: A New Class of Azole-Type β-Glucosidase Inhibitor. J. Am. Chem. Soc..

[43] Nahoum V., Roux G., Anton V., Rougé P., Puigserver A., Bischoff H (2000). Crystal structures of human pancreatic α-amylase in complex with carbohydrate and proteinaceous inhibitors. Biochem. J..

[44] Russell R.J., Haire L.F., Stevens D.J., Collins P.J., Lin Y.P., Blackburn G.M (2006). The structure of H5N1 avian influenza neuraminidase suggests new opportunities for drug design. Nature.

[45] Artola M., Wu L., Ferraz M.J., Kuo C.-L., Raich L., Breen I.Z (2017). 1,6-Cyclophellitol cyclosulfates: a new class of irreversible glycosidase inhibitor. ACS Cent. Sci..

[46] Vocadlo D.J., Davies G.J (2008). Mechanistic insights into glycosidase chemistry. Curr. Opin. Chem. Biol..

[47] Zechel D.L., Withers S.G (2000). Glycosidase mechanisms: anatomy of a finely tuned catalyst. Acc. Chem. Res..

[48] Kötzler M.P., Hancock S.M., Withers S.G (2014). In eLS.

[49] Torronen A., Rouvinen J (1995). Structural comparison of Two major endo-1,4-Xylanases from trichoderma reesei. Biochemistry.

[50] Chavas L.M.G., Tringali C., Fusi P., Venerando B., Tettamanti G., Kato R (2005). Crystal Structure of the Human Cytosolic Sialidase Neu2. Journal of Biological Chemistry.

[51] Zechel D.L., Boraston A.B., Gloster T., Boraston C.M., Macdonald J.M., Tilbrook D.M.G (2003). Iminosugar glycosidase inhibitors: structural and thermodynamic dissection of the binding of isofagomine and 1-deoxynojirimycin to beta-glucosidases. J. Am. Chem. Soc..

[52] Nurizzo D., Turkenburg J.P., Charnock S.J., Roberts S.M., Dodson E.J., McKie V.A (2002). Cellvibrio japonicus alpha-L-arabinanase 43A has a novel five-blade beta-propeller fold. Nat. Struct. Biol..

[53] Lee J., Paetzel M (2011). Structure of the catalytic domain of glucoamylase from Aspergillus niger. Acta Crystallogr. Sect. F Struct. Biol. Cryst. Commun..

[54] Heinz D.W., Baase W.A., Dahlquist F.W., Matthews B.W (1993). How amino-acid insertions are allowed in an alpha-helix of T4 lysozyme. Nature.

[55] Safran J., Tabi W., Ung V., Lemaire A., Habrylo O., Bouckaert J (2023). Plant polygalacturonase structures specify enzyme dynamics and processivities to fine-tune cell wall pectins. Plant Cell.

[56] Vavricka C.J., Liu Y., Kiyota H., Sriwilaijaroen N., Qi J., Tanaka K (2013). Influenza neuraminidase operates via a nucleophilic mechanism and can be targeted by covalent inhibitors. Nat. Commun..

[57] Mark B.L., Vocadlo D.J., Knapp S., Triggs-Raine B.L., Withers S.G., James M.N.G (2001). Crystallographic evidence for substrate-assisted catalysis in a bacterial β-hexosaminidase. Journal of Biological Chemistry.

[58] Sobala L.F., Speciale G., Zhu S., Raich L., Sannikova N., Thompson A.J (2020). An epoxide Intermediate in glycosidase catalysis. ACS Cent. Sci..

[59] Yip V.L.Y., Withers S.G (2006). Family 4 glycosidases carry out efficient hydrolysis of thioglycosides by an alpha,beta-elimination mechanism. Angew. Chem. Int. Ed. Engl..

[60] Yip V.L.Y., Thompson J., Withers S.G (2007). Mechanism of GlvA from bacillus subtilis: a detailed kinetic analysis of a 6-phospho-alpha-glucosidase from glycoside hydrolase family 4. Biochemistry.

[61] Jongkees S.A.K., Withers S.G (2014). Unusual enzymatic glycoside cleavage mechanisms. Acc. Chem. Res..

[62] Kaur A., Pickles I.B., Sharma M., Madeido Soler N., Scott N.E., Pidot S.J (2023). Widespread family of NAD^+^-dependent sulfoquinovosidases at the gateway to sulfoquinovose catabolism. J. Am. Chem. Soc..

[63] Breton C., Fournel-Gigleux S., Palcic M.M (2012). Recent structures, evolution and mechanisms of glycosyltransferases. Curr. Opin. Struct. Biol..

[64] Alexander J.A.N., Locher K.P (2023). Emerging structural insights into C-type glycosyltransferases. Curr. Opin. Struct. Biol..

[65] Bloch J.S., Pesciullesi G., Boilevin J., Nosol K., Irobalieva R.N., Darbre T (2020). Structure and mechanism of the ER-based glucosyltransferase ALG6. Nature.

[66] Patenaude S.I., Seto N.O.L., Borisova S.N., Szpacenko A., Marcus S.L., Palcic M.M (2002). The structural basis for specificity in human ABO(H) blood group biosynthesis. Nat. Struct. Biol..

[67] Martinez-Fleites C., Proctor M., Roberts S., Bolam D.N., Gilbert H.J., Davies G.J (2006). Insights into the Synthesis of Lipopolysaccharide and Antibiotics through the Structures of Two Retaining Glycosyltransferases from Family GT4. Chem. Biol..

[68] Darby J.F., Gilio A.K., Piniello B., Roth C., Blagova E., Hubbard R.E (2020). Substrate engagement and catalytic mechanisms of N-acetylglucosaminyltransferase V. ACS Catal..

[69] Morgan J.L.W., Strumillo J., Zimmer J (2013). Crystallographic snapshot of cellulose synthesis and membrane translocation. Nature.

[70] Morgan J.L.W., McNamara J.T., Fischer M., Rich J., Chen H.-M., Withers S.G (2016). Observing cellulose biosynthesis and membrane translocation in crystallo. Nature.

[71] Purushotham P., Ho R., Zimmer J (2020). Architecture of a catalytically active homotrimeric plant cellulose synthase complex. Science.

[72] Maloney F.P., Kuklewicz J., Corey R.A., Bi Y., Ho R., Mateusiak L (2022). Structure, substrate recognition and initiation of hyaluronan synthase. Nature.

[73] Ramakrishnan B., Balaji P.V., Qasba P.K (2002). Crystal structure of beta1,4-galactosyltransferase complex with UDP-Gal reveals an oligosaccharide acceptor binding site. J. Mol. Biol..

[74] Ramakrishnan B., Qasba P.K (2001). Crystal structure of lactose synthase reveals a large conformational change in its catalytic component, the beta1,4-galactosyltransferase-I. J. Mol. Biol..

[75] Boix E., Swaminathan G.J., Zhang Y., Natesh R., Brew K., Acharya K.R (2001). Structure of UDP complex of UDP-galactose:β-galactoside-α-1,3-galactosyltransferase at 1.53-Å resolution reveals a conformational change in the catalytically important C terminus. Journal of Biological Chemistry.

[76] Nagae M., Kizuka Y., Mihara E., Kitago Y., Hanashima S., Ito Y (2018). Structure and mechanism of cancer-associated N-acetylglucosaminyltransferase-V. Nat. Commun..

[77] Bloch J.S., John A., Mao R., Mukherjee S., Boilevin J., Irobalieva R.N (2023). Structure, sequon recognition and mechanism of tryptophan C-mannosyltransferase. Nat. Chem. Biol..

[78] Lee S.S., Hong S.Y., Errey J.C., Izumi A., Davies G.J., Davis B.G (2011). Mechanistic evidence for a front-side, SNi-type reaction in a retaining glycosyltransferase. Nat. Chem. Biol..

[79] Ardèvol A., Rovira C (2011). The molecular mechanism of enzymatic glycosyl transfer with retention of configuration: evidence for a short-lived oxocarbenium-like species. Angew. Chem. Int. Ed. Engl..

[80] Bilyard M.K., Bailey H.J., Raich L., Gafitescu M.A., Machida T., Iglésias-Fernández J (2018). Palladium-mediated enzyme activation suggests multiphase initiation of glycogenesis. Nature.

[81] Lira-Navarrete E., Iglesias-Fernández J., Zandberg W.F., Compañón I., Kong Y., Corzana F (2014). Substrate-guided front-face reaction revealed by combined structural snapshots and metadynamics for the polypeptide N-acetylgalactosaminyltransferase 2. Angew. Chem. Int. Ed. Engl..

[82] Doyle L., Ovchinnikova O.G., Huang B.-S., Forrester T.J.B., Lowary T.L., Kimber M.S (2023). Mechanism and linkage specificities of the dual retaining β-Kdo glycosyltransferase modules of KpsC from bacterial capsule biosynthesis. J. Biol. Chem..

[83] Sagiroglugil M., Liao Q., Planas A., Rovira C (2024). Formation of a Covalent Adduct in Retaining β‐Kdo Glycosyl‐Transferase WbbB via Substrate‐Mediated Proton Relay. ChemCatChem.

[84] Zhang L., Zhao Y., Gao Y., Wu L., Gao R., Zhang Q (2020). Structures of cell wall arabinosyltransferases with the anti-tuberculosis drug ethambutol. Science.

[85] Petrou V.I., Herrera C.M., Schultz K.M., Clarke O.B., Vendome J., Tomasek D (2016). Structures of aminoarabinose transferase arnT suggest A molecular basis for lipid A glycosylation. Science.

[86] Bai L., Kovach A., You Q., Kenny A., Li H (2019). Structure of the eukaryotic protein O-mannosyltransferase Pmt1-Pmt2 complex. Nat. Struct. Mol. Biol..

[87] Sjodt M., Brock K., Dobihal G., Rohs P.D.A., Green A.G., Hopf T.A (2018). Structure of the peptidoglycan polymerase RodA resolved by evolutionary coupling analysis. Nature.

[88] Ashraf K.U., Nygaard R., Vickery O.N., Erramilli S.K., Herrera C.M., McConville T.H (2022). Structural basis of lipopolysaccharide maturation by the O-antigen ligase. Nature.

[89] Lizak C., Gerber S., Numao S., Aebi M., Locher K.P (2011). X-ray structure of a bacterial oligosaccharyltransferase. Nature.

[90] Yang X., Qian K (2017). Protein O-GlcNAcylation: emerging mechanisms and functions. Nat. Rev. Mol. Cell Biol..

[91] Shcherbakova A., Preller M., Taft M.H., Pujols J., Ventura S., Tiemann B (2019). C-mannosylation supports folding and enhances stability of thrombospondin repeats. Elife.

[92] Holdener B.C., Haltiwanger R.S (2019). Protein O-fucosylation: structure and function. Curr. Opin. Struct. Biol..

[93] Housley M.P., Rodgers J.T., Udeshi N.D., Kelly T.J., Shabanowitz J., Hunt D.F (2008). O-GlcNAc regulates FoxO activation in response to glucose. J. Biol. Chem..

[94] Golks A., Tran T.-T.T., Goetschy J.F., Guerini D (2007). Requirement for O-linked N-acetylglucosaminyltransferase in lymphocytes activation. EMBO J..

[95] Dentin R., Hedrick S., Xie J., Yates J., Montminy M (2008). Hepatic glucose sensing via the CREB coactivator CRTC2. Science.

[96] Chu C.-S., Lo P.-W., Yeh Y.-H., Hsu P.-H., Peng S.-H., Teng Y.-C (2014). O-GlcNAcylation regulates EZH2 protein stability and function. Proc. Natl. Acad. Sci. U.S.A..

[97] Gambetta M.C., Oktaba K., Müller J (2009). Essential role of the glycosyltransferase sxc/Ogt in polycomb repression. Science.

[98] Kuntz D.A., Tarling C.A., Withers S.G., Rose D.R (2008). Structural analysis of Golgi alpha-mannosidase II inhibitors identified from a focused glycosidase inhibitor screen. Biochemistry.

[99] Kadirvelraj R., Yang J.-Y., Sanders J.H., Liu L., Ramiah A., Prabhakar P.K (2018). Human *N*-acetylglucosaminyltransferase II substrate recognition uses a modular architecture that includes a convergent exosite. Proc. Natl. Acad. Sci. U.S.A..

[100] Harrus D., Harduin-Lepers A., Glumoff T (2020). Unliganded and CMP-Neu5Ac bound structures of human α-2,6-sialyltransferase ST6Gal I at high resolution. J. Struct. Biol..

[101] Gordon R.D., Sivarajah P., Satkunarajah M., Ma D., Tarling C.A., Vizitiu D (2006). X-ray crystal structures of rabbit N-acetylglucosaminyltransferase I (GnT I) in complex with donor substrate analogues. J. Mol. Biol..

[102] Gastinel L.N., Cambillau C., Bourne Y (1999). Crystal structures of the bovine beta4galactosyltransferase catalytic domain and its complex with uridine diphosphogalactose. EMBO J..

[103] Xiang Y., Karaveg K., Moremen K.W (2016). Substrate recognition and catalysis by GH47 α-mannosidases involved in Asn-linked glycan maturation in the mammalian secretory pathway. Proc. Natl. Acad. Sci. U.S.A..

[104] Warfield K.L., Alonzi D.S., Hill J.C., Caputo A.T., Roversi P., Kiappes J.L (2020). Targeting endoplasmic reticulum α-glucosidase I with a single-dose iminosugar treatment protects against lethal influenza and dengue virus infections. J. Med. Chem..

[105] Su C.-C., Lyu M., Zhang Z., Miyagi M., Huang W., Taylor D.J (2023). High-resolution structural-omics of human liver enzymes. Cell Rep..

[106] Leisico F., Omeiri J., Le Narvor C., Beaudouin J., Hons M., Fenel D (2022). Structure of the human heparan sulfate polymerase complex EXT1-EXT2. Nat. Commun..

[107] Mycroft-West C.J., Abdelkarim S., Duyvesteyn H.M.E., Gandhi N.S., Skidmore M.A., Owens R.J (2024). Structural and mechanistic characterization of bifunctional heparan sulfate N-deacetylase-N-sulfotransferase 1. Nat. Commun..

[108] Debarnot C., Monneau Y.R., Roig-Zamboni V., Delauzun V., Le Narvor C., Richard E (2019). Substrate binding mode and catalytic mechanism of human heparan sulfate d-glucuronyl C5 epimerase. Proc. Natl. Acad. Sci. U.S.A..

[109] Xu Y., Moon A.F., Xu S., Krahn J.M., Liu J., Pedersen L.C (2017). Structure Based Substrate Specificity Analysis of Heparan Sulfate 6-O-Sulfotransferases. ACS Chem. Biol..

[110] Bethea H.N., Xu D., Liu J., Pedersen L.C (2008). Redirecting the substrate specificity of heparan sulfate 2-O-sulfotransferase by structurally guided mutagenesis. Proc. Natl. Acad. Sci. U.S.A..

[111] Wilson L.F.L., Dendooven T., Hardwick S.W., Echevarría-Poza A., Tryfona T., Krogh K.B.R.M (2022). The structure of EXTL3 helps to explain the different roles of bi-domain exostosins in heparan sulfate synthesis. Nat. Commun..

[112] Li H., Chapla D., Amos R.A., Ramiah A., Moremen K.W., Li H (2023). Structural basis for heparan sulfate co-polymerase action by the EXT1-2 complex. Nat. Chem. Biol..

[113] Xu D., Moon A.F., Song D., Pedersen L.C., Liu J (2008). Engineering sulfotransferases to modify heparan sulfate. Nat. Chem. Biol..

[114] Wander R., Kaminski A.M., Xu Y., Pagadala V., Krahn J.M., Pham T.Q (2021). Deciphering the substrate recognition mechanisms of the heparan sulfate 3-*O*-sulfotransferase-3. RSC Chem. Biol..

[115] Moon A.F., Xu Y., Woody S.M., Krahn J.M., Linhardt R.J., Liu J (2012). Dissecting the substrate recognition of 3-O-sulfotransferase for the biosynthesis of anticoagulant heparin. Proc. Natl. Acad. Sci. U.S.A..

[116] Dunphy W.G., Brands R., Rothman J.E (1985). Attachment of terminal N-acetylglucosamine to asparagine-linked oligosaccharides occurs in central cisternae of the golgi stack. Cell.

[117] Roth J., Berger E.G (1982). Immunocytochemical localization of galactosyltransferase in HeLa cells: codistribution with thiamine pyrophosphatase in trans-Golgi cisternae. J. Cell Biol..

[118] Rabouille C., Hui N., Hunte F., Kieckbusch R., Berger E.G., Warren G (1995). Mapping the distribution of Golgi enzymes involved in the construction of complex oligosaccharides. J. Cell. Sci..

[119] Nilsson T., Hoe M.H., Slusarewicz P., Rabouille C., Watson R., Hunte F (1994). Kin recognition between medial Golgi enzymes in HeLa cells. EMBO J..

[120] Jungmann J., Munro S (1998). Multi-protein complexes in the cis golgi of saccharomyces cerevisiae with alpha-1,6-mannosyltransferase activity. EMBO J..

[121] Schoberer J., Liebminger E., Botchway S.W., Strasser R., Hawes C (2013). Time-resolved fluorescence imaging reveals differential interactions of N-glycan processing enzymes across the Golgi stack in planta. Plant Physiol..

[122] Hassinen A., Pujol F.M., Kokkonen N., Pieters C., Kihlström M., Korhonen K (2011). Functional organization of Golgi N- and O-glycosylation pathways involves pH-dependent complex formation that is impaired in cancer cells. J. Biol. Chem..

[123] McCormick C., Duncan G., Goutsos K.T., Tufaro F (2000). The putative tumor suppressors EXT1 and EXT2 form a stable complex that accumulates in the Golgi apparatus and catalyzes the synthesis of heparan sulfate. Proc. Natl. Acad. Sci. U.S.A..

[124] Presto J., Thuveson M., Carlsson P., Busse M., Wilén M., Eriksson I (2008). Heparan sulfate biosynthesis enzymes EXT1 and EXT2 affect NDST1 expression and heparan sulfate sulfation. Proc. Natl. Acad. Sci. U.S.A..

[125] Préchoux A., Halimi C., Simorre J.-P., Lortat-Jacob H., Laguri C (2015). C5-epimerase and 2-O-sulfotransferase associate in vitro to generate contiguous epimerized and 2-O-sulfated heparan sulfate domains. ACS Chem. Biol..

[126] Pinhal M.A., Smith B., Olson S., Aikawa J., Kimata K., Esko J.D (2001). Enzyme interactions in heparan sulfate biosynthesis: uronosyl 5-epimerase and 2-O-sulfotransferase interact in vivo. Proc. Natl. Acad. Sci. U.S.A..

[127] Lidholt K., Kjellén L., Lindahl U (1989). Biosynthesis of heparin. Relationship between the polymerization and sulphation processes. Biochem. J..

[128] Esko J.D., Selleck S.B (2002). Order out of chaos: assembly of ligand binding sites in heparan sulfate. Annu. Rev. Biochem..

[129] Hassinen A., Kellokumpu S (2014). Organizational interplay of Golgi N-glycosyltransferases involves organelle microenvironment-dependent transitions between enzyme homo- and heteromers. J. Biol. Chem..

[130] Tie H.C., Mahajan D., Lu L (2022). Visualizing intra-Golgi localization and transport by side-averaging Golgi ministacks. J. Cell Biol..

[131] Tie H.C., Ludwig A., Sandin S., Lu L (2018). The spatial separation of processing and transport functions to the interior and periphery of the golgi stack. Elife.

[132] Yagi H., Tateo S., Saito T., Ohta Y., Nishi E., Obitsu S (2024). Deciphering the sub-Golgi localization of glycosyltransferases via 3D super-resolution imaging. Cell Struct. Funct..

[133] Meneghetti M.C.Z., Deboni P., Palomino C.M.V., Braga L.P., Cavalheiro R.P., Viana G.M (2021). ER-Golgi dynamics of HS-modifying enzymes via vesicular trafficking is a critical prerequisite for the delineation of HS biosynthesis. Carbohydr. Polym..

[134] Harada A., Kunii M., Kurokawa K., Sumi T., Kanda S., Zhang Y (2024). Dynamic movement of the Golgi unit and its glycosylation enzyme zones. Nat. Commun..

[135] Rice W.J., Cheng A., Noble A.J., Eng E.T., Kim L.Y., Carragher B (2018). Routine determination of ice thickness for cryo-EM grids. J. Struct. Biol..

[136] Mangala Prasad V., Leaman D.P., Lovendahl K.N., Croft J.T., Benhaim M.A., Hodge E.A (2022). Cryo-ET of Env on intact HIV virions reveals structural variation and positioning on the Gag lattice. Cell.

[137] Yao H., Song Y., Chen Y., Wu N., Xu J., Sun C (2020). Molecular Architecture of the SARS-CoV-2 Virus. Cell.

[138] Graham M., Zhang P (2023). Cryo-electron tomography to study viral infection. Biochem. Soc. Trans..

[139] Huang Q.J., Song K., Xu C., Bolon D.N.A., Wang J.P., Finberg R.W (2022). Quantitative structural analysis of influenza virus by cryo-electron tomography and convolutional neural networks. Structure.

[140] Fowler S.L., Behr T.S., Turkes E., Cauhy P.M., Foiani M.S., Schaler A (2023). Tau filaments are tethered within brain extracellular vesicles in Alzheimer’s disease. bioRxiv.

[141] O’Reilly F.J., Xue L., Graziadei A., Sinn L., Lenz S., Tegunov D (2020). In-cell architecture of an actively transcribing-translating expressome. Science.

[142] Xue L., Lenz S., Zimmermann-Kogadeeva M., Tegunov D., Cramer P., Bork P (2022). Visualizing translation dynamics at atomic detail inside a bacterial cell. Nature.

[143] Foster H.E., Ventura Santos C., Carter A.P (2022). A cryo-ET survey of microtubules and intracellular compartments in mammalian axons. J. Cell Biol..

[144] Fukuda Y., Beck F., Plitzko J.M., Baumeister W (2017). In situ structural studies of tripeptidyl peptidase II (TPPII) reveal spatial association with proteasomes. Proc. Natl. Acad. Sci. U.S.A..

[145] Mageswaran S.K., Yang W.Y., Chakrabarty Y., Oikonomou C.M., Jensen G.J (2021). A cryo-electron tomography workflow reveals protrusion-mediated shedding on injured plasma membrane. Sci. Adv..

[146] Mesman R.J (2013). A novel method for high-pressure freezing of adherent cells for frozen hydrated sectioning and CEMOVIS. J. Struct. Biol..

[147] Schaffer M., Mahamid J., Engel B.D., Laugks T., Baumeister W., Plitzko J.M (2017). Optimized cryo-focused ion beam sample preparation aimed at in situ structural studies of membrane proteins. J. Struct. Biol..

[148] Dumoux M., Glen T., Smith J.L.R., Ho E.M.L., Perdigão L.M.A., Pennington A (2023). Cryo-plasma FIB/SEM volume imaging of biological specimens. Elife.

[149] Berger C., Dumoux M., Glen T., Yee N.B.-Y., Mitchels J.M., Patáková Z (2023). Plasma FIB milling for the determination of structures in situ. Nat. Commun..

[150] Klumpe S., Fung H.K., Goetz S.K., Zagoriy I., Hampoelz B., Zhang X (2021). A modular platform for automated cryo-FIB workflows. Elife.

[151] Schiøtz O.H., Kaiser C.J.O., Klumpe S., Morado D.R., Poege M., Schneider J (2024). Serial Lift-Out: sampling the molecular anatomy of whole organisms. Nat. Methods.

[152] Schaffer M., Pfeffer S., Mahamid J., Kleindiek S., Laugks T., Albert S (2019). A cryo-FIB lift-out technique enables molecular-resolution cryo-ET within native Caenorhabditis elegans tissue. Nat. Methods.

[153] Nguyen H.T.D., Perone G., Klena N., Vazzana R., Kaluthantrige Don F., Silva M (2024). Serialized on-grid lift-in sectioning for tomography (SOLIST) enables a biopsy at the nanoscale. Nat. Methods.

[154] Gilbert M.A.G., Fatima N., Jenkins J., O’Sullivan T.J., Schertel A., Halfon Y (2024). CryoET of β-amyloid and tau within postmortem Alzheimer’s disease brain. Nature.

[155] Glynn C., Smith J.L.R., Case M., Csöndör R., Katsini A., Sanita M.E Charting the molecular landscape of neuronal organisation within the hippocampus using cryo electron tomography. Biochemistry.

[156] Kuba J., Mitchels J., Hovorka M., Erdmann P., Berka L., Kirmse R (2021). Advanced cryo-tomography workflow developments - correlative microscopy, milling automation and cryo-lift-out. J. Microsc..

[157] Gorelick S., Buckley G., Gervinskas G., Johnson T.K., Handley A., Caggiano M.P (2019). PIE-scope, integrated cryo-correlative light and FIB/SEM microscopy. Elife.

[158] Bieber A., Capitanio C., Schiøtz O., Smeets M., Fenzke J., Erdmann P (2021). Precise 3D-correlative FIB-milling of biological samples using METEOR, an integrated cryo-CLEM imaging system. Microsc. Microanal..

[159] Baker L.A., Rubinstein J.L (2010). Radiation damage in electron cryomicroscopy. Meth. Enzymol..

[160] Hagen W.J.H., Wan W., Briggs J.A.G (2017). Implementation of a cryo-electron tomography tilt-scheme optimized for high resolution subtomogram averaging. J. Struct. Biol..

[161] Nakane T., Kotecha A., Sente A., McMullan G., Masiulis S., Brown P.M.G.E (2020). Single-particle cryo-EM at atomic resolution. Nature.

[162] Schwartz O., Axelrod J.J., Campbell S.L., Turnbaugh C., Glaeser R.M., Müller H (2019). Laser phase plate for transmission electron microscopy. Nat. Methods.

[163] Danev R., Buijsse B., Khoshouei M., Plitzko J.M., Baumeister W (2014). Volta potential phase plate for in-focus phase contrast transmission electron microscopy. Proc. Natl. Acad. Sci. U.S.A..

[164] Zheng S., Wolff G., Greenan G., Chen Z., Faas F.G.A., Bárcena M (2022). AreTomo: An integrated software package for automated marker-free, motion-corrected cryo-electron tomographic alignment and reconstruction. J. Struct. Biol..

[165] Tegunov D., Xue L., Dienemann C., Cramer P., Mahamid J (2021). Multi-particle cryo-EM refinement with M visualizes ribosome-antibiotic complex at 3.5 Å in cells. Nat. Methods.

[166] Tegunov D., Cramer P (2019). Real-time cryo-electron microscopy data preprocessing with Warp. Nat. Methods.

[167] Goetz S.K., Mattausch A., Stojanovska F., Zimmerli C.E., Toro-Nahuelpan M (2023). Convolutional networks for supervised mining of molecular patterns within cellular context. Nat. Methods.

[168] Pennington A., King O.N.F., Tun W.M., Ho E.M.L., Luengo I., Darrow M.C (2022). Survos 2: accelerating annotation and segmentation for large volumetric bioimage workflows across modalities and scales. Front. Cell Dev. Biol..

[169] Buchholz T.-O., Krull A., Shahidi R., Pigino G., Jékely G., Jug F (2019).

[170] Lamm L., Zufferey S., Righetto R.D., Wietrzynski W., Yamauchi K.A., Burt A MemBrain v2: an end-to-end tool for the analysis of membranes in cryo-electron tomography. Bioinformatics.

[171] Liu Y.-T., Zhang H., Wang H., Tao C.-L., Bi G.-Q., Zhou Z.H (2022). Isotropic reconstruction for electron tomography with deep learning. Nat. Commun..

[172] McCafferty C.L., Klumpe S., Amaro R.E., Kukulski W., Collinson L., Engel B.D (2024). Integrating cellular electron microscopy with multimodal data to explore biology across space and time. Cell.

[173] Nogales E., Mahamid J (2024). Bridging structural and cell biology with cryo-electron microscopy. Nature.

[174] Berger C., Premaraj N., Ravelli R.B.G., Knoops K., López-Iglesias C., Peters P.J (2023). Cryo-electron tomography on focused ion beam lamellae transforms structural cell biology. Nat. Methods.

[175] Bykov Y.S., Schaffer M., Dodonova S.O., Albert S., Plitzko J.M., Baumeister W (2017). The structure of the COPI coat determined within the cell. Elife.

[176] Ruiz-Canada C., Kelleher D.J., Gilmore R (2009). Cotranslational and posttranslational N-glycosylation of polypeptides by distinct mammalian OST isoforms. Cell.

[177] Pfeffer S., Brandt F., Hrabe T., Lang S., Eibauer M., Zimmermann R (2012). Structure and 3D arrangement of endoplasmic reticulum membrane-associated ribosomes. Structure.

[178] Pfeffer S., Dudek J., Gogala M., Schorr S., Linxweiler J., Lang S (2014). Structure of the mammalian oligosaccharyl-transferase complex in the native ER protein translocon. Nat. Commun..

[179] Braunger K., Pfeffer S., Shrimal S., Gilmore R., Berninghausen O., Mandon E.C (2018). Structural basis for coupling protein transport and N-glycosylation at the mammalian endoplasmic reticulum. Science.

[180] Gemmer M., Chaillet M.L., Cuevas Arenas R., Vismpas D., Gröllers-Mulderij M (2023). Visualization of translation and protein biogenesis at the ER membrane. Nature.

[181] Nguyen D., Stutz R., Schorr S., Lang S., Pfeffer S., Freeze H.H (2018). Proteomics reveals signal peptide features determining the client specificity in human TRAP-dependent ER protein import. Nat. Commun..

[182] Sjostrand F.S., Hanzon V (1954). Ultrastructure of Golgi apparatus of exocrine cells of mouse pancreas. Exp. Cell Res..

[183] Dalton A.J., Felix M.D (1956). A comparative study of the Golgi complex. J. Biophys. Biochem. Cytol..

[184] Farquhar M.G., Rinehart J.F (1954). Cytologic alterations in the anterior pituitary gland following thyroidectomy: an electron microscope study. Endocrinology.

[185] Bouchet-Marquis C., Starkuviene V., Grabenbauer M (2008). Golgi apparatus studied in vitreous sections. J. Microsc..

[186] Engel B.D., Schaffer M., Albert S., Asano S., Plitzko J.M., Baumeister W (2015). In situ structural analysis of Golgi intracisternal protein arrays. Proc. Natl. Acad. Sci. U.S.A..

[187] Zhang X., Carter S.D., Singla J., White K.L., Butler P.C., Stevens R.C (2020). Visualizing insulin vesicle neighborhoods in β cells by cryo-electron tomography. Sci. Adv..

[188] Lee C., Goldberg J (2010). Structure of coatomer cage proteins and the relationship among COPI, COPII, and clathrin vesicle coats. Cell.

[189] Faini M., Prinz S., Beck R., Schorb M., Riches J.D., Bacia K (2012). The structures of COPI-coated vesicles reveal alternate coatomer conformations and interactions. Science.

[190] Beck M., Lučić V., Förster F., Baumeister W., Medalia O (2007). Snapshots of nuclear pore complexes in action captured by cryo-electron tomography. Nature.

[191] Roos N., Cyrklaff M., Cudmore S., Blasco R., Krijnse-Locker J., Griffiths G (1996). A novel immunogold cryoelectron microscopic approach to investigate the structure of the intracellular and extracellular forms of vaccinia virus. EMBO J..

[192] Yi H., Strauss J.D., Ke Z., Alonas E., Dillard R.S., Hampton C.M (2015). Native immunogold labeling of cell surface proteins and viral glycoproteins for cryo-electron microscopy and cryo-electron tomography applications. J. Histochem. Cytochem..

[193] Jiang J., Cheong K.Y., Falkowski P.G., Dai W (2021). Integrating on-grid immunogold labeling and cryo-electron tomography to reveal photosystem II structure and spatial distribution in thylakoid membranes. J. Struct. Biol..

[194] Azubel M., Carter S.D., Weiszmann J., Zhang J., Jensen G.J., Li Y (2019). FGF21 trafficking in intact human cells revealed by cryo-electron tomography with gold nanoparticles. Elife.

[195] Young L.N., Sherrard A., Zhou H., Shaikh F., Hutchings J., Riggi M (2024). ExoSloNano: Multi-Modal Nanogold Tags for identification of Macromolecules in Live Cells & Cryo-Electron Tomograms. bioRxiv.

[196] Muyldermans S (2013). Nanobodies: natural single-domain antibodies. Annu. Rev. Biochem..

[197] Berger C., Ravelli R.B.G., López-Iglesias C., Peters P.J (2021). Endocytosed nanogold fiducials for improved in-situ cryo-electron tomography tilt-series alignment. J. Struct. Biol..

[198] Mercogliano C.P., DeRosier D.J (2007). Concatenated metallothionein as a clonable gold label for electron microscopy. J. Struct. Biol..

[199] Wang Q., Mercogliano C.P., Löwe J (2011). A ferritin-based label for cellular electron cryotomography. Structure.

[200] Clarke N.I., Royle S.J (2018). FerriTag is a new genetically-encoded inducible tag for correlative light-electron microscopy. Nat. Commun..

[201] Silvester E., Vollmer B., Pražák V., Vasishtan D., Machala E.A., Whittle C (2021). DNA origami signposts for identifying proteins on cell membranes by electron cryotomography. Cell.

[202] Fung H.K.H., Hayashi Y., Salo V.T., Babenko A., Zagoriy I., Brunner A (2023). Genetically encoded multimeric tags for subcellular protein localization in cryo-EM. Nat. Methods.

[203] Cruz-León S., Majtner T., Hoffmann P.C., Kreysing J.P., Kehl S., Tuijtel M.W (2024). High-confidence 3D template matching for cryo-electron tomography. Nat. Commun..

[204] Szabo G.V., Burg T.P (2024). Time Resolved Cryo‐Correlative Light and Electron Microscopy. Adv. Funct. Mater..

[205] Fuest M., Nocera G.M., Modena M.M., Riedel D., Mejia Y.X., Burg T.P (2018). Cryofixation during live-imaging enables millisecond time-correlated light and electron microscopy. J. Microsc..

